# Supplementary material for: Glycoprotein 130 Antagonism Counteracts Metabolic and Inflammatory Alterations to Enhance Right Ventricle Function in Pulmonary Artery Banded Pigs
Source: bioRxiv. 2025 Jan 22:2025.01.20.633954. Preprint. [Version 1] doi: 10.1101/2025.01.20.633954 (PMC11785131; doi:10.1101/2025.01.20.633954)

bioRxiv preprint doi: https://doi.org/10.1101/2025.01.20.633954; this version posted January 22, 2025. The copyright holder for this preprint (which was not certified by peer review) is the author/funder, who has granted bioRxiv a license to display the preprint in perpetuity. It is made available under aCC-BY 4.0 International license.

Supplemental Table 1: Complete cMRI data. Data are presented as mean ± standard deviation or median and [interquartile range]. Subscript “i” indicates value indexed to final pig weight.

	Control (n=5)	PAB-VEH (n=5)	PAB-SC144 (n=5)
**RA EDV_i (mL/kg)**	1.1 [0.9-1.1]	1.5 [1.4-1.9]	1.4 [0.9-2.3]
**RA ESV_i (mL/kg)**	0.4 [0.4-0.5]	0.9 [0.8-1.1]	0.7 [0.5-1.6]
**RA SVi (mL/kg)**	0.6 ± 0.1	0.6 ± 0.2	0.6 ± 0.2
**RA EF (%)**	60 [54-63]	35 [34-46]	46 [33-53]
**RA SV/ESV**	1.5 [1.2-1.7]	0.5 [0.5-0.9]	0.9 [0.5-1.1]
**RV EDV_i (mL/kg)**	2.4 ± 0.5	4.9 ± 1.8	3.6 ± 1.3
**RV ESV_i (mL/kg)**	1.0 ± .2	3.9 ± 1.9	2.3 ± 1.0
**RV SV_i (mL/kg)**	1.4 ± 0.3	1.0 ± 0.2	1.3 ± 0.5
**RVEF (%)**	59 ± 4	22 ± 10	37 ± 6
**RV $Mass_i$ (g/kg)**	0.8 ± 0.2	1.8 ± 0.4	1.5 ± 0.4
**RV SV/ESV**	1.5 [1.3-1.7]	0.3 [0.1-0.5]	0.5 [0.5-0.8]
**LV EDV_i (mL/kg)**	3.3 ± 0.5	3.0 ± 0.2	2.4 ± 0.3
**LV ESV_i (mL/kg)**	1.7 ± 0.4	1.8 ± 0.4	1.3 ± 0.3
**LV SV_i (mL/kg)**	1.6 ± 0.2	1.1 ± 0.3	1.1 ± 0.1
**LV EF**	48.2 ± 5	39 ± 11	47 ± 6
**RVEDV/LVEDV**	0.7 [0.7-0.8]	1.4 [1.2-2.4]	1.3 [1.1-1.9]
**TR Severity**	NA	1.8 ± 0.4	1.5 ± 0.4
**Proximal PA CSA**	NA	2.8 ± 0.6	2.6 ± 0.4
**PA-Band CSA**	NA	0.3 ± 0.1	0.4 ± 0.1
**% Stenosis**	NA	89 ± 2	86 ± 5

bioRxiv preprint doi: https://doi.org/10.1101/2025.01.20.633954; this version posted January 22, 2025. The copyright holder for this preprint (which was not certified by peer review) is the author/funder, who has granted bioRxiv a license to display the preprint in perpetuity. It is made available under aCC-BY 4.0 International license.

Supplemental Figure 1: **Neither PAB nor SC144 significantly affected fibrosis**. Representative microscopy images of RV sections stained with picrosirius red to evaluate fibrosis. Fibrosis was similar between all three groups (control: 2.7±0.7%, PAB-Veh 2.6±0.8%, PAB-SC144: 2.4±0.9%)

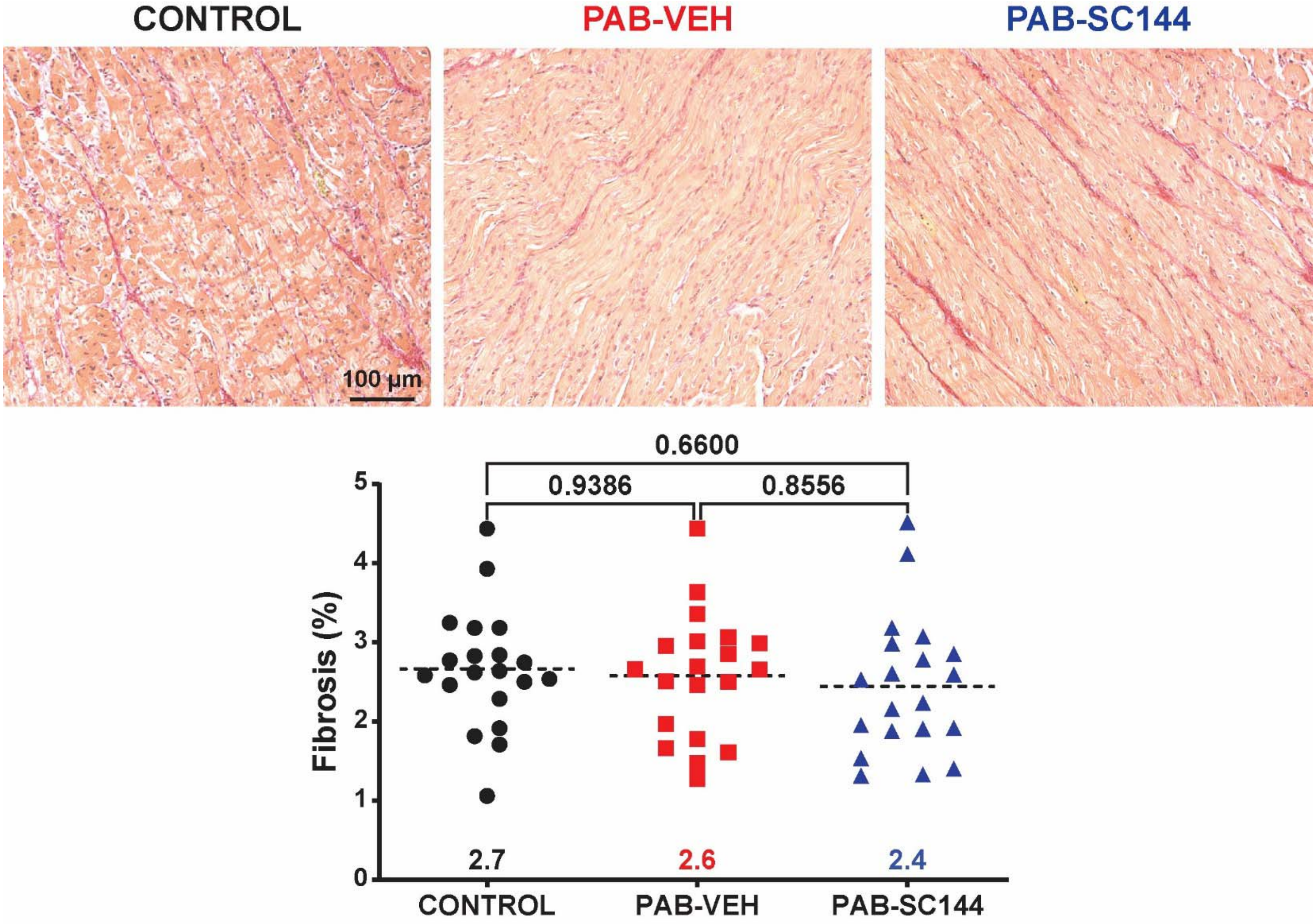

bioRxiv preprint doi: https://doi.org/10.1101/2025.01.20.633954; this version posted January 22, 2025. The copyright holder for this preprint (which was not certified by peer review) is the author/funder, who has granted bioRxiv a license to display the preprint in perpetuity. It is made available under aCC-BY 4.0 International license.

Supplemental Figure 2: **SC144 counteracted metabolic gene expression dysregulation in cardiomyocytes**. (**A**) Pathways enriched in cardiomyocyte subgroup 3 (CM3), found mostly in PAB-Veh RVs, were related to inflammation and mTOR signaling (italics, red star). (**B**) Metabolic pathways, including oxidative phosphorylation (italics, red star), were elevated in CM4, which were identified at similar proportions in control and PAB-SC144.

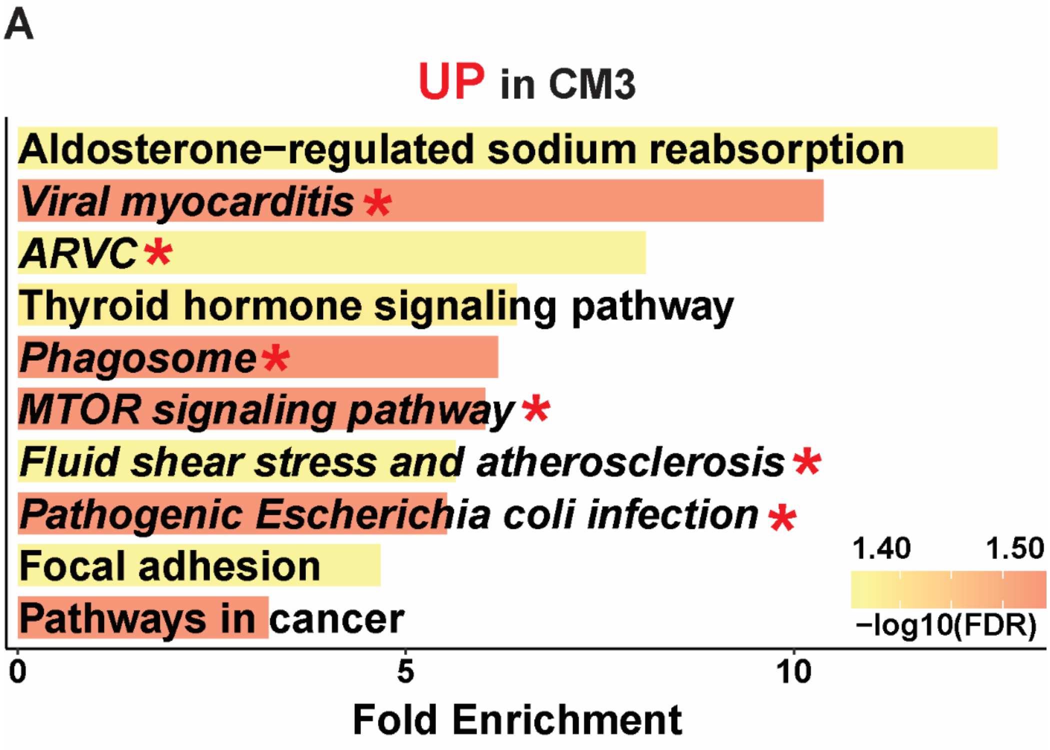

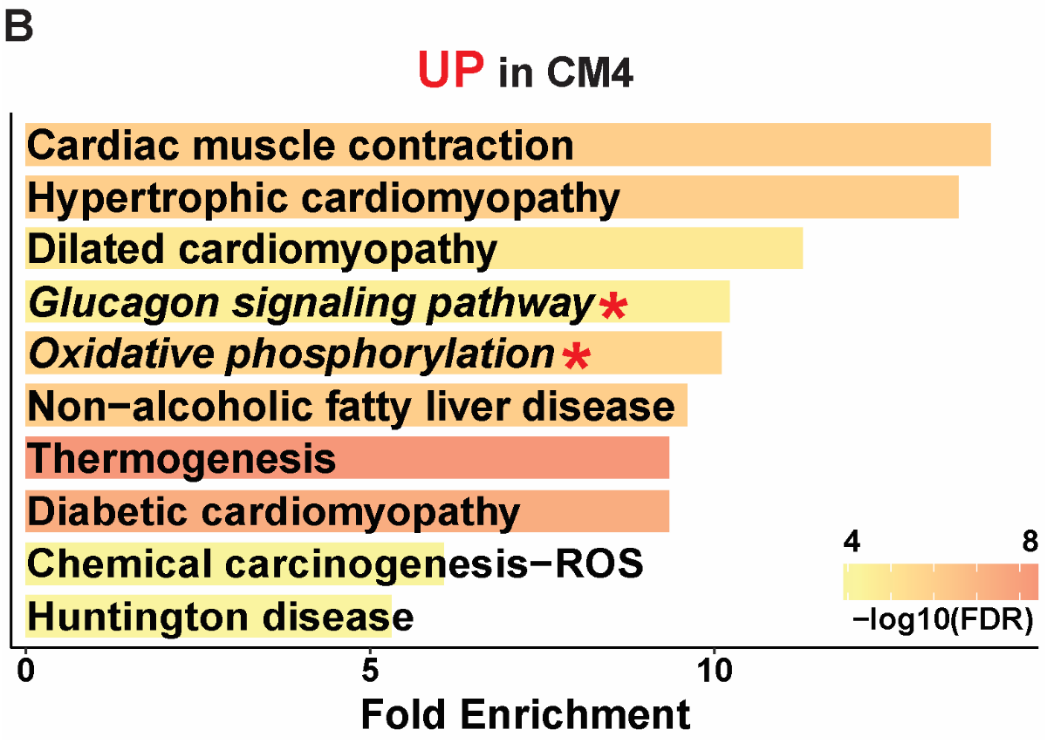

bioRxiv preprint doi: https://doi.org/10.1101/2025.01.20.633954; this version posted January 22, 2025. The copyright holder for this preprint (which was not certified by peer review) is the author/funder, who has granted bioRxiv a license to display the preprint in perpetuity. It is made available under aCC-BY 4.0 International license.

Supplemental Figure 3: **Pro-inflammatory pathways and predicted communication from immune cells are increased in PAB-Veh immune cells**. Compared to control, (**A**) pathways that regulate macrophage differentiation, survival and proliferation (italics, red star) were suppressed in PAB-Veh, and the pro-inflammatory pathway toll-like receptor signaling was enriched in PAB-Veh macrophages. (**B**) Anti-inflammatory pathways were reduced in PAB-Veh lymphocytes relative to control, and oxidative phosphorylation was increased. CellChat communication analysis showing (**C**) predicted communication from macrophages to endothelial cells, pericytes, and cardiomyocytes was higher (red) in PAB-Veh macrophages compared to control, but (**D**) reduced (blue) in PAB-SC144 immune cells compared to control. (**E**) Compared to PAB-Veh, PAB-SC144 immune cells had reduced predicted signaling.

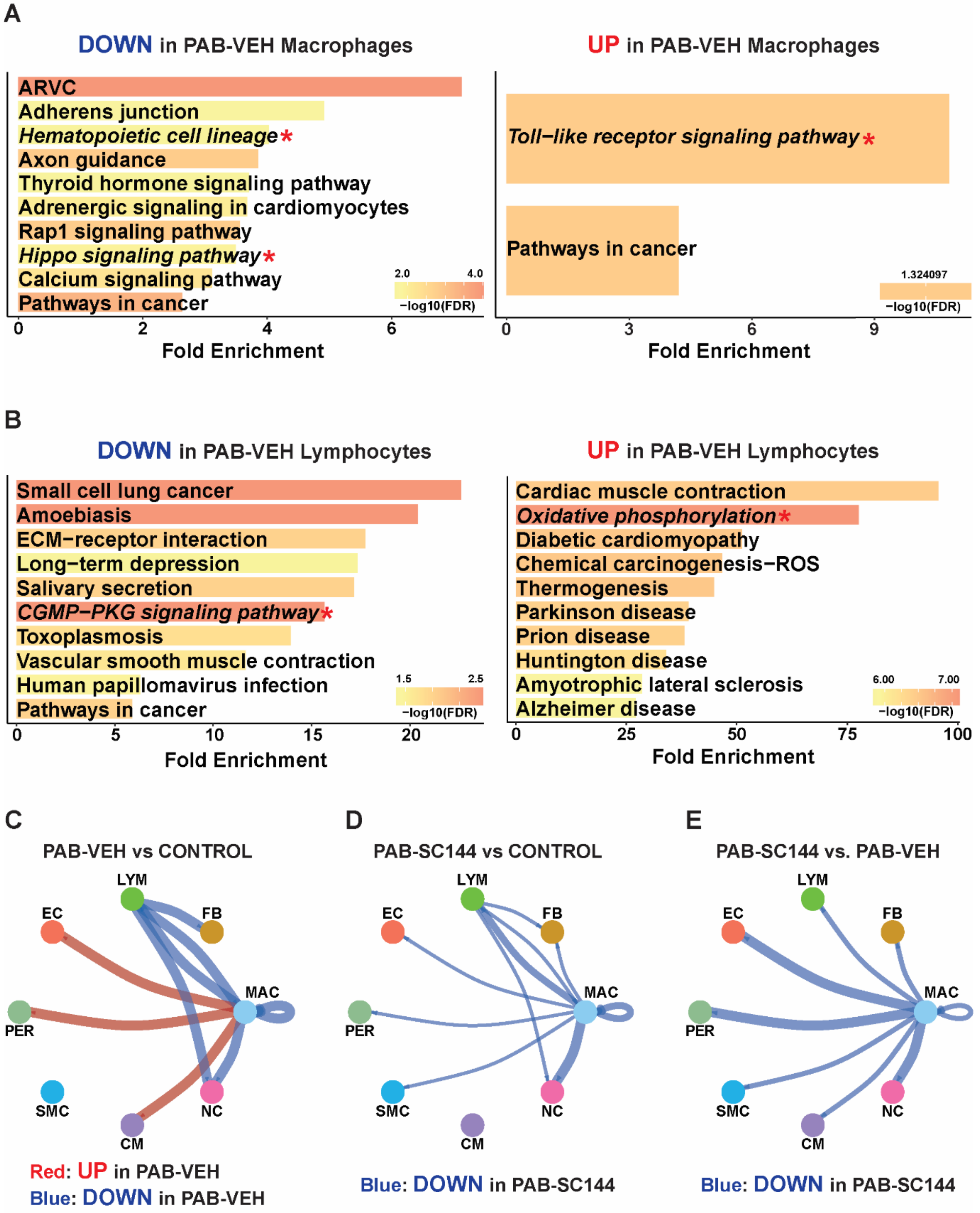

bioRxiv preprint doi: https://doi.org/10.1101/2025.01.20.633954; this version posted January 22, 2025. The copyright holder for this preprint (which was not certified by peer review) is the author/funder, who has granted bioRxiv a license to display the preprint in perpetuity. It is made available under aCC-BY 4.0 International license.

Supplemental Table 2: Grouped abundances of phosphorylated mTOR (p-mTOR). p-mTOR was elevated in PAB-Veh, but normalized by SC144.

	Control	PAB-VEH	PAB-SC144
**p-mTOR (AU)**	7405	84960	4624

bioRxiv preprint doi: https://doi.org/10.1101/2025.01.20.633954; this version posted January 22, 2025. The copyright holder for this preprint (which was not certified by peer review) is the author/funder, who has granted bioRxiv a license to display the preprint in perpetuity. It is made available under aCC-BY 4.0 International license.

Supplemental Figure 4: **GP130 antagonism with SC144 is predicted to alter metabolic kinases.** (**A**) Several polo like kinases (PLK, red italics) which antagonize mTORC1 were estimated to be diminished in PAB-Veh compared to control. PLK5 was not in the Coral Database and is therefore not visualized on the kinome map (red star). (**B**) Multiple pyruvate dehydrogenase kinases (PDK, red italics) which support glucose utilization over fatty acid oxidation were predicted to be reduced in PAB-SC144 compared to control.

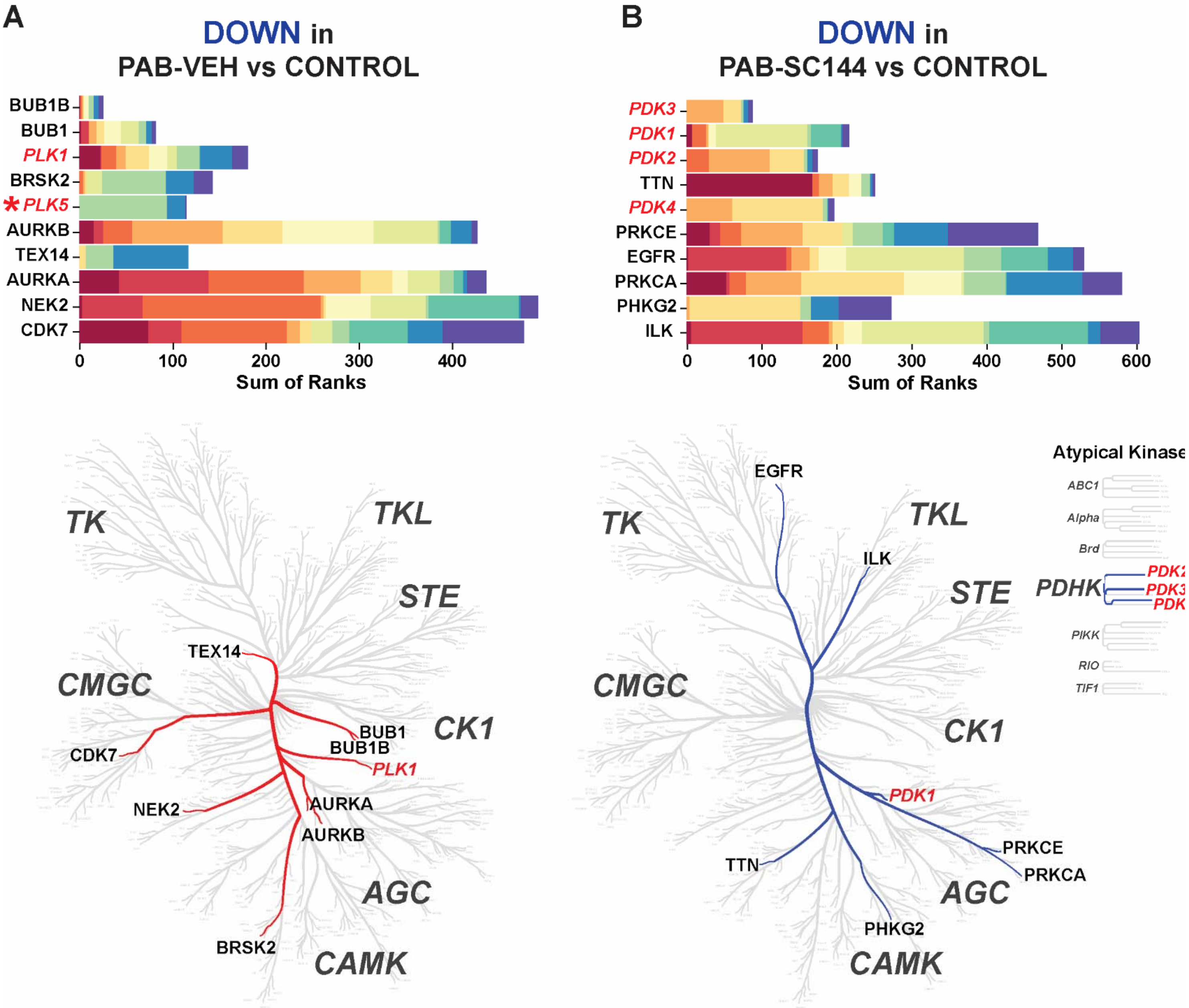

bioRxiv preprint doi: https://doi.org/10.1101/2025.01.20.633954; this version posted January 22, 2025. The copyright holder for this preprint (which was not certified by peer review) is the author/funder, who has granted bioRxiv a license to display the preprint in perpetuity. It is made available under aCC-BY 4.0 International license.

Supplemental Figure 5: **PAB did not affect RV or serum carnitine levels**. RV (0.9, [0.8-1.0]) and serum (0.9, [0.7-1.0]) carnitine levels are similar between all 3 groups, but serum carnitine was slightly elevated in PAB-SC144 pigs. Kruskal-Wallis ANOVA with Dunn post hoc test used.

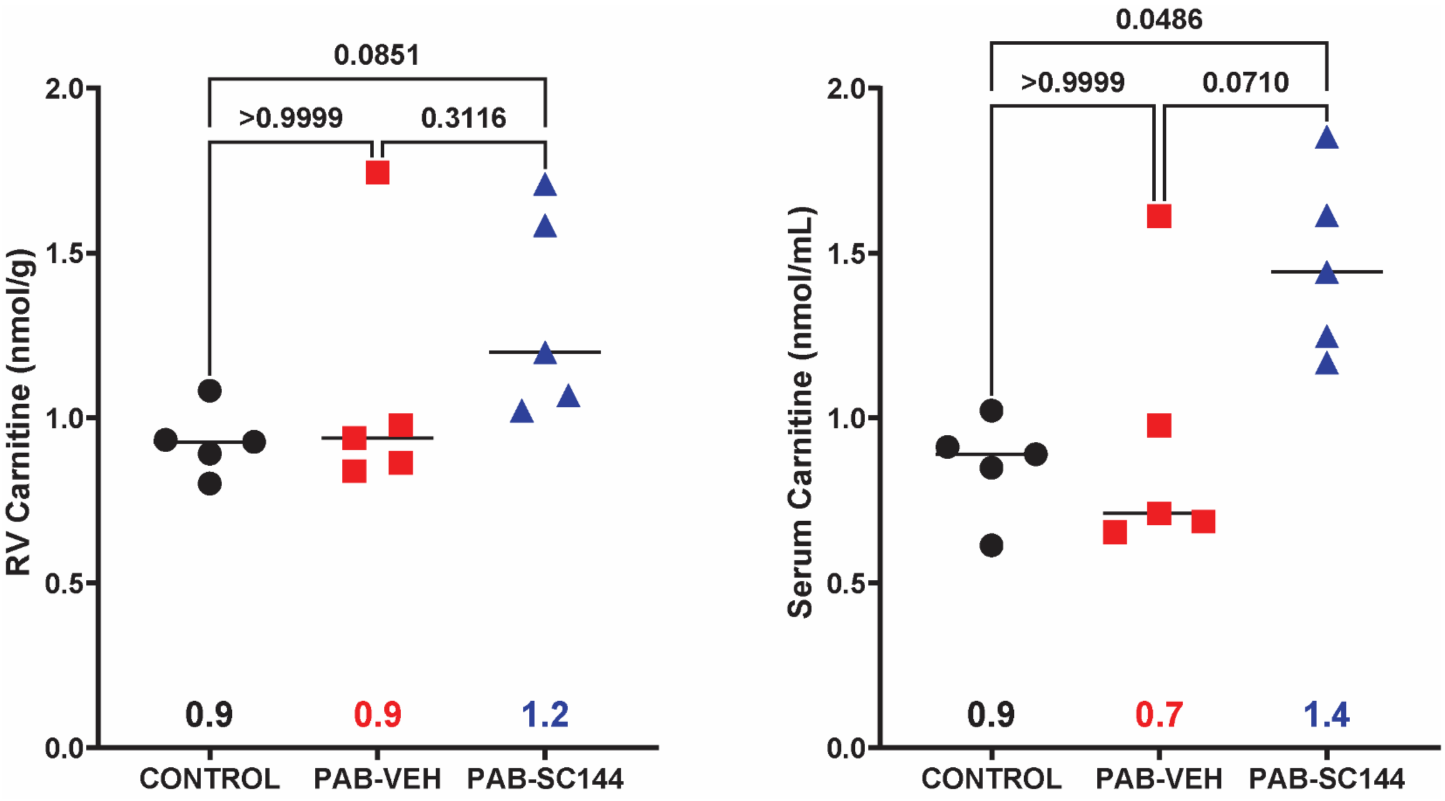

bioRxiv preprint doi: https://doi.org/10.1101/2025.01.20.633954; this version posted January 22, 2025. The copyright holder for this preprint (which was not certified by peer review) is the author/funder, who has granted bioRxiv a license to display the preprint in perpetuity. It is made available under aCC-BY 4.0 International license.

Supplemental Figure 6: **Serum lipidomics and metabolomics reveal changes in RV fat metabolism were not likely due to reduced systemic lipid or fatty acid availability.** (**A**) TAG, (**B**) DAG, and (**C**) MAG species were mostly elevated in PAB-Veh serum compared to control and PAB-SC144. Serum (**D**) long and (**E**) medium chain fatty acids were increased in PAB-Veh, and acylcarnitines were similarly elevated in PAB-SC144 and control serum. Many amino acid metabolism intermediates in the (**G**) histidine, (**H**) arginine and proline, and (**I**) methionine, cysteine and taurine pathways were increased in both PAB-Veh and PAB-SC144. Scale bars show relative changes between groups.

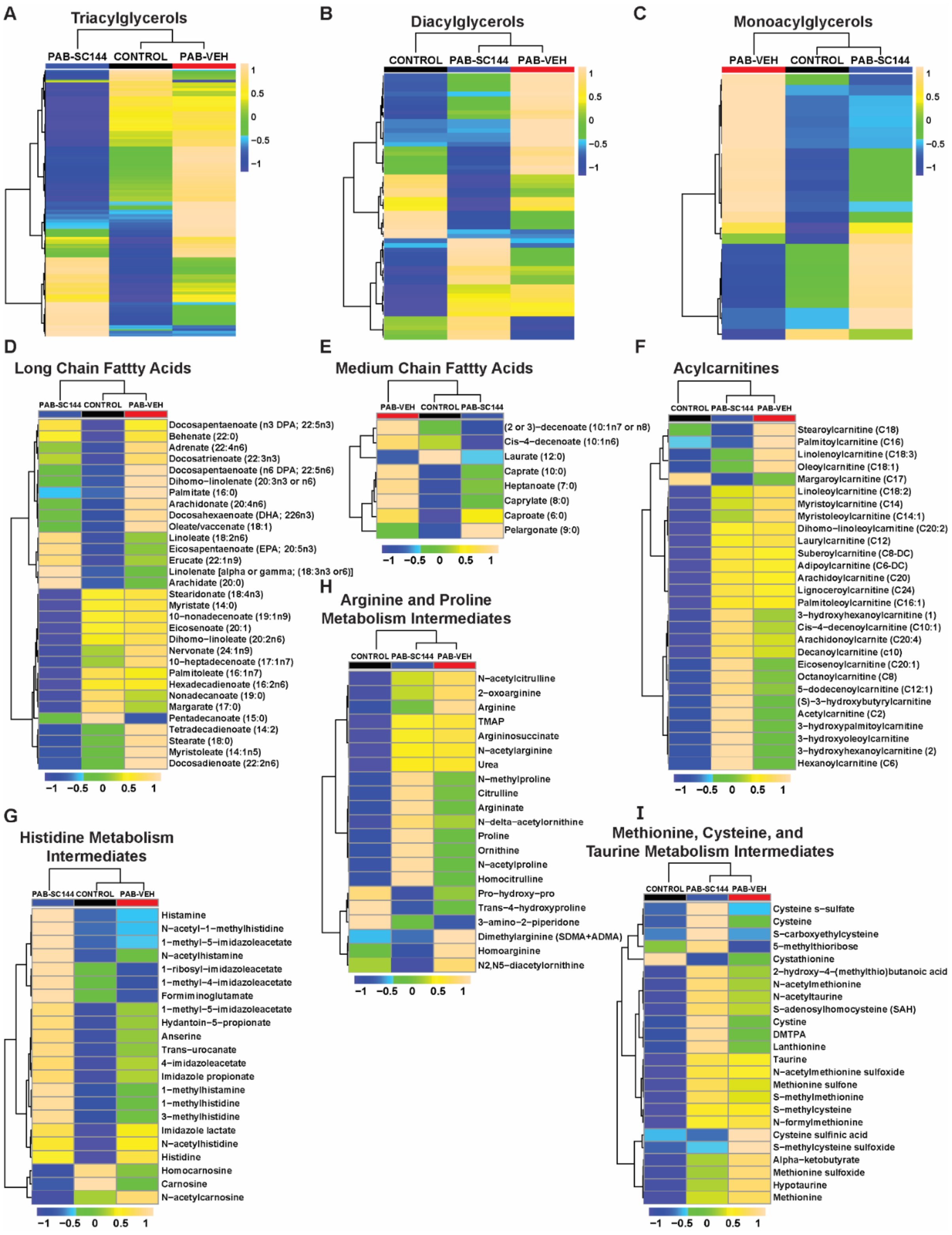

bioRxiv preprint doi: https://doi.org/10.1101/2025.01.20.633954; this version posted January 22, 2025. The copyright holder for this preprint (which was not certified by peer review) is the author/funder, who has granted bioRxiv a license to display the preprint in perpetuity. It is made available under aCC-BY 4.0 International license.

Supplemental Figure 7: **Ceramide species are not elevated in PAB-Veh RVs compared to control or PAB-SC144.** Scale bar shows relative changes between groups

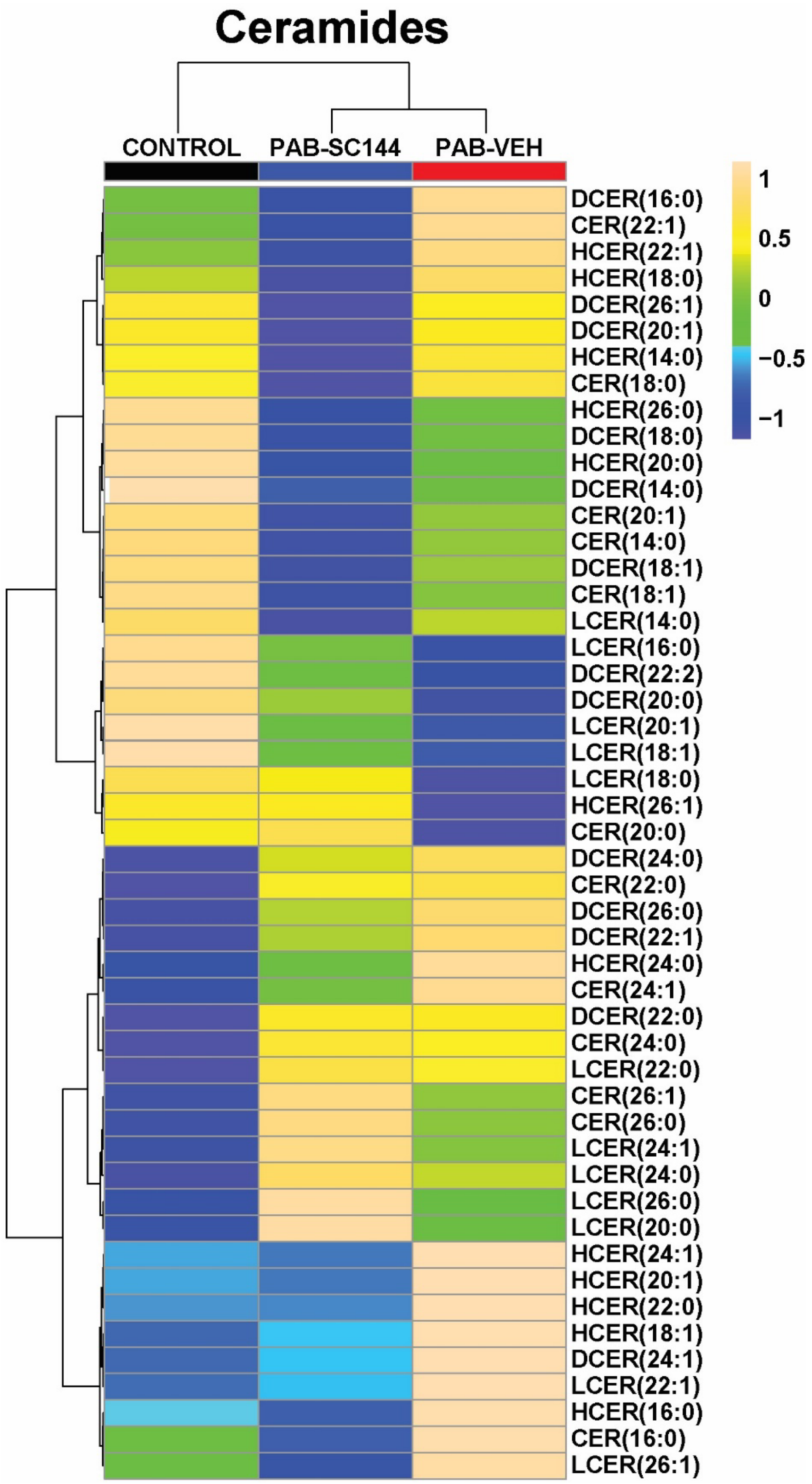

bioRxiv preprint doi: https://doi.org/10.1101/2025.01.20.633954; this version posted January 22, 2025. The copyright holder for this preprint (which was not certified by peer review) is the author/funder, who has granted bioRxiv a license to display the preprint in perpetuity. It is made available under aCC-BY 4.0 International license.

Supplemental Figure 8: **GP130 inhibition modulates endothelial cell and pericyte biology**. (**A**) Pathway analysis of PAB-Veh DEGs compared to control identify endothelial cell repair mechanisms may be reduced, but replication is not altered. (**B**) Pathways that promote proper pericyte-endothelial cell interactions during angiogenesis are reduced in PAB-Veh pericytes. (**C**) CellChat analysis identified 3 ligand receptor pairs (red boxes) only enriched in PAB-SC144 vascular cells, not PAB-Veh that contribute to endothelial cell-pericyte stability.

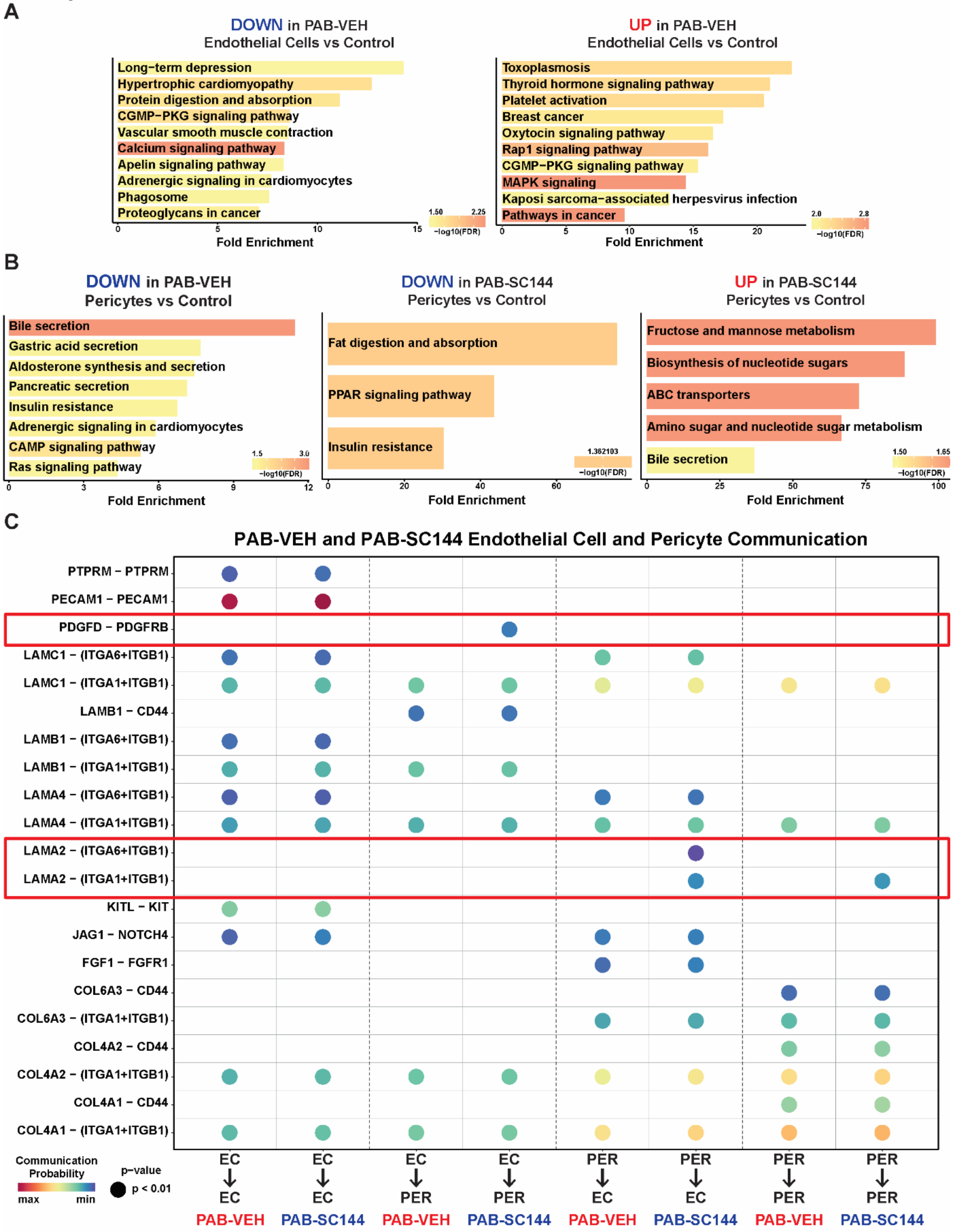

bioRxiv preprint doi: https://doi.org/10.1101/2025.01.20.633954; this version posted January 22, 2025. The copyright holder for this preprint (which was not certified by peer review) is the author/funder, who has granted bioRxiv a license to display the preprint in perpetuity. It is made available under aCC-BY 4.0 International license.

Supplemental Figure 9: **GP130 receptor expression across all cell types**. Besides GP130, the leukemia inhibitory factor receptor (LIFR), IL11 receptor (IL11RA), and oncostatin M receptor (OSMR) had the highest expression. The IL6 receptor (IL6R) and ciliary neurotrophic factor receptor (CNTFR) had lower expression in the RV.

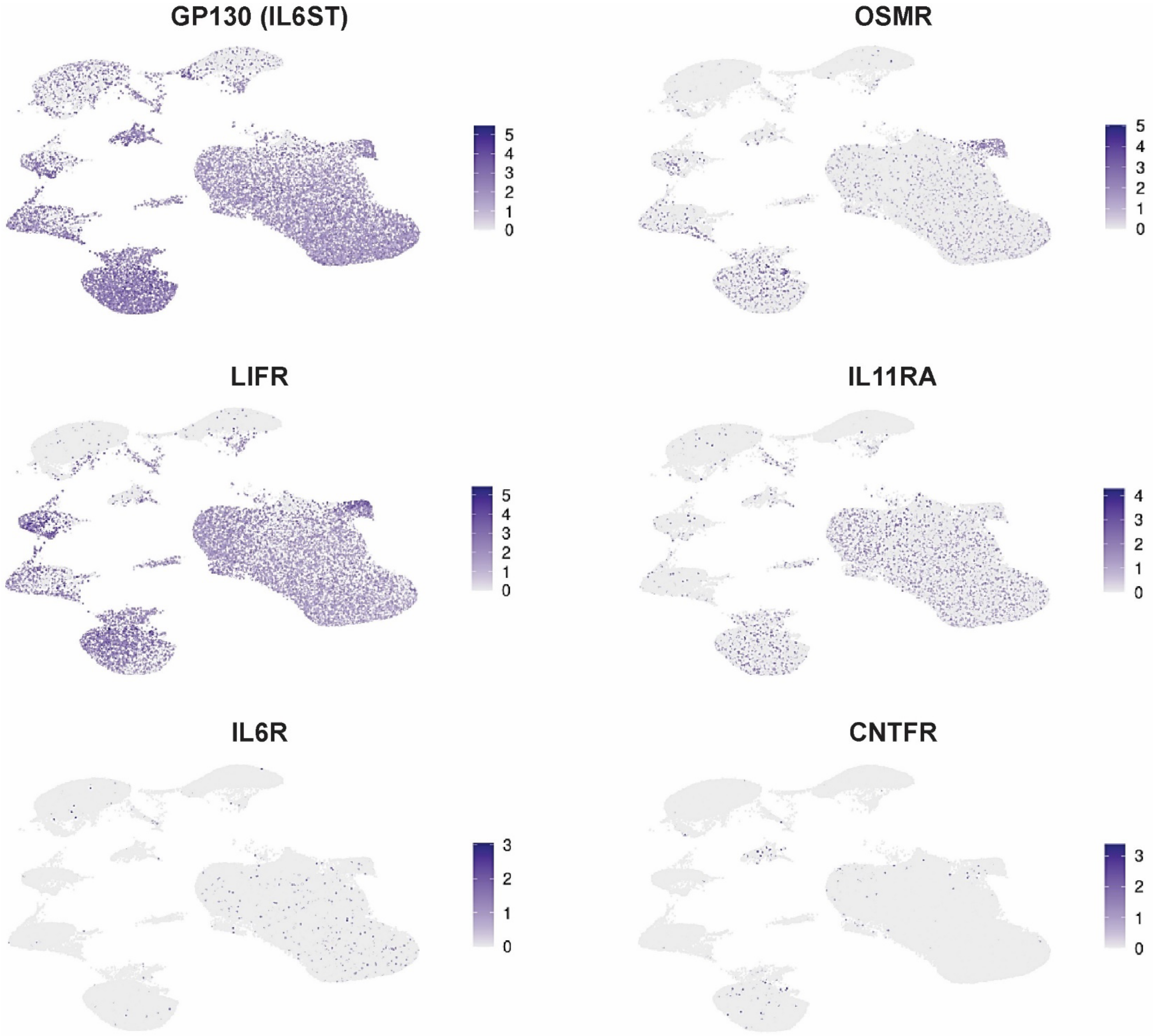

bioRxiv preprint doi: https://doi.org/10.1101/2025.01.20.633954; this version posted January 22, 2025. The copyright holder for this preprint (which was not certified by peer review) is the author/funder, who has granted bioRxiv a license to display the preprint in perpetuity. It is made available under aCC-BY 4.0 International license.

Supplemental Figure 10: **SC144 normalized RV tubulin abundances, but PAB did not cause a significant increase in these proteins**. Representative western blots and quantification of alpha(α)-, beta(β)-, and detyrosinated α-tubulin.

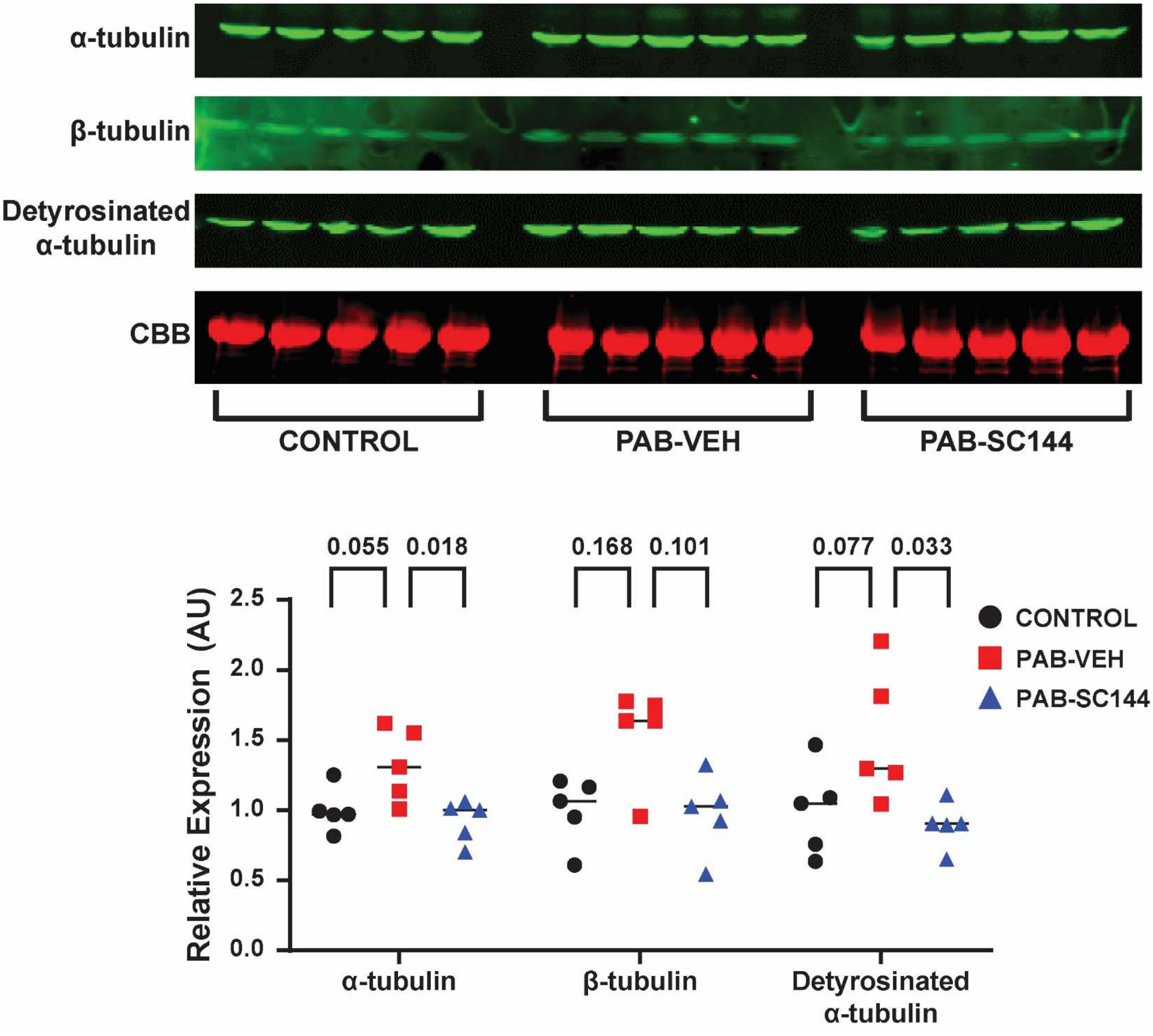

Supplement: Supplement 2 [file NIHPP2025.01.20.633954v1-supplement-2.pdf]
